# Supplementary material for: The Transcription Factors WRKY41 and WRKY53 Mediate Early Flowering Induced by the Novel Plant Growth Regulator Guvermectin in Arabidopsis thaliana
Source: Int J Mol Sci. 2023 May 8;24(9):8424. doi: 10.3390/ijms24098424 (PMC10178944; doi:10.3390/ijms24098424)
Supplement: Supplementary file 1 [file ijms-24-08424-s001.zip › ijms-2317309-supplementary/ijms-2317309-Supplementary Figures.pdf]

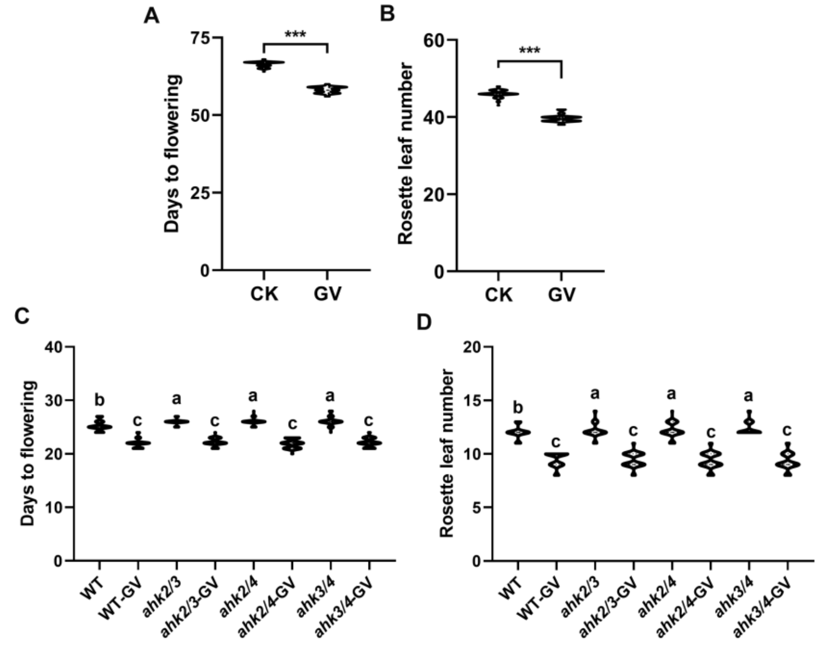


**Figure S1 GV accelerates flowering in *Arabidopsis*.** (A, B) Flowering phenotypes associated with GV treatment and CK as assessed by DTF (A) and RLN (B) grown under short day conditions. 7-week-old plants were sprayed with 50 mg $L^{-1}$ GV and CK (0 mg $L^{-1}$ GV). A significant difference analysis was Student's t-test (***, $p < 0.001$). (C, D) Flowering phenotypes of WT and three cytokinin receptor mutants ahk2/3, ahk2/4, and ahk3/4 associated with GV treatment as assessed by DTF (C) and RLN (D) grown under long day conditions. 2-week-old plants were sprayed with 50 mg l-1 GV and 0 mg $L^{-1}$ GV treatment was used as a control. CK, control (treated with 0 mg $L^{-1}$ GV). GV, 50 mg $L^{-1}$ GV treatment. Different letters above the bars indicate statistically significant differences (adjusted $P < 0.05$, one-way ANOVA). Three biological replicates were counted with similar results. Values are expressed as means ± SD (n=15).

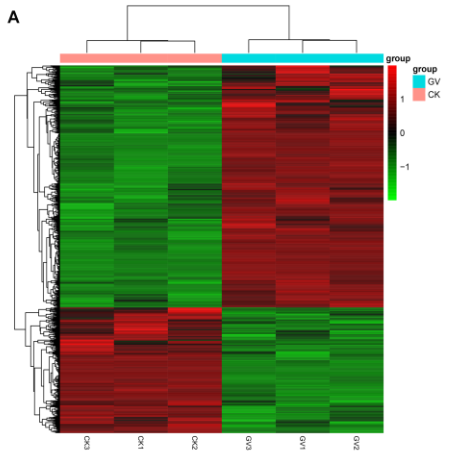


B

| Gene id | Gene_name | $log_2$FoldChange |
|---|---|---|
| 824257 | BCS1 | 1.798955 |
| 839470 | CYP703A2 | 6.600049 |
| 821902 | ERF1 | 3.880447 |
| 824763 | JRG21 | 2.812200 |
| 837305 | LBD1 | 2.292989 |
| 815949 | PR1 | 1.801018 |

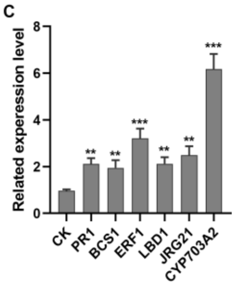


**Figure S2 Analysis and validation of transcriptome data**. (A) Clustering heat map of differentially expressed genes induced by GV. (B) The genes induced significantly by GV in our RNA-seq data. (C) Transcripts detection of genes in (A) by RT-qPCR. A significant difference analysis was Student's *t*-test (**, $p < 0.01$; ***, $p < 0.001$)

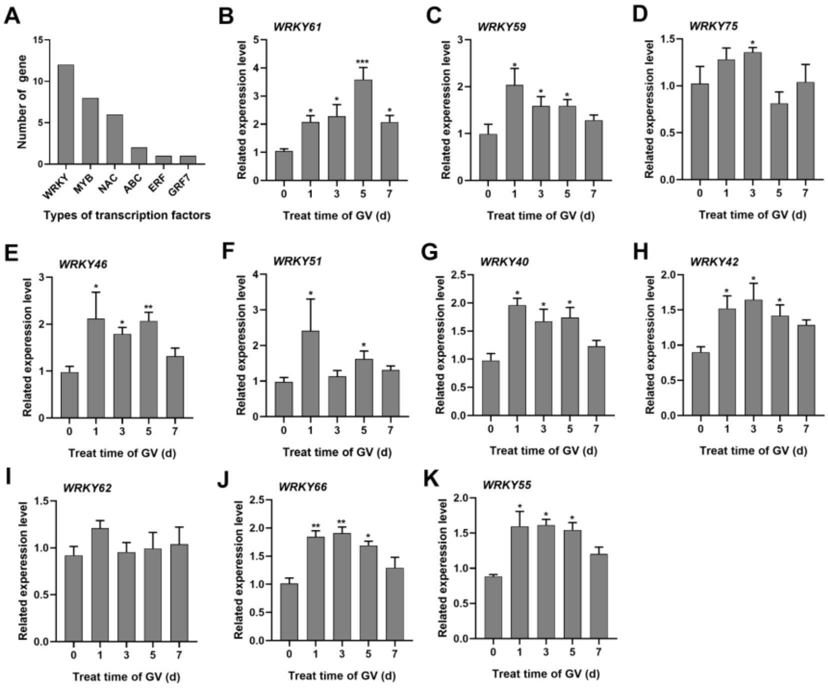


**Figure S3 WRKY TFs were induced by GV**. (A) The number of major transcription factor genes induced by GV. (B-K) The related transcript levels of *WRKY61* (B), *WRKY59* (C), *WRKY75* (D), *WRKY46* (E), *WRKY51* (F), *WRKY40* (G), *WRKY42* (H), *WRKY62* (I), *WRKY66* (J), and *WRKY55* (K) at 0, 1, 3, 5, and 7 d after 50 mg $L^{-1}$ and 0 mg $L^{-1}$ GV treatment. The WRKY TFs induced by GV at $\log_2$FoldChange ≥ 1.5 were included in this analysis. Each experiment was repeated three times with similar results. Values are expressed as means ± SD (n=3). A significant difference analysis was Student's *t*-test (*, $p < 0.05$; **, $p < 0.01$, ***, $p < 0.001$).

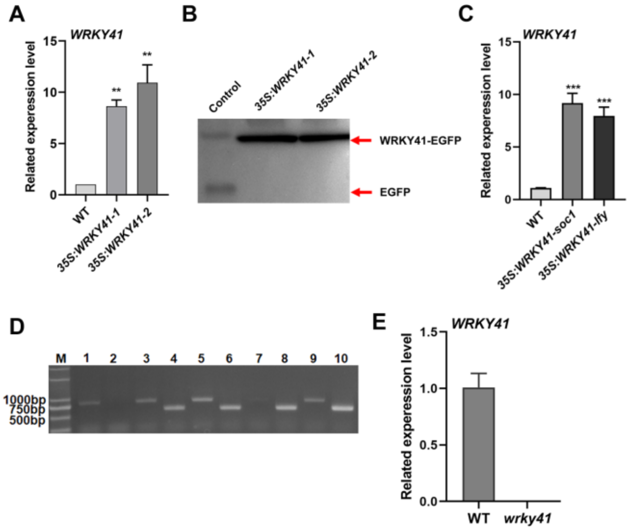


**Figure S4 Validation of *WRKY41* transgenic lines and T-DNA insertion mutants**. (A) The related transcript levels of *WRKY41* in two *35S:WRKY41* lines. Similar results were obtained from three replicates. (B) Western blot analysis of two *35S:WRKY41*-GFP lines. Control, EGFP protein. (C) The related transcript levels of *WRKY41* in two *35S:WRKY41-soc1* and *35S:WRKY41-lfy* lines. (D) Validation of *WRKY41* T-DNA insertion mutants using the three primers. LP+BP included 1, 3, 5, 7, and 9 Lanes. BP+RP included 2, 4, 6, 8, and 10 Lanes. LP, RP: Left, Right genomic primer. BP: T-DNA border primer. M, DNA marker. 1-2, WT. 3-10, *wrky41* lines. Lanes 7 and 8 represent the homozygous lines; lanes 3 4, 5 6, 9 10 represent the heterozygous lines. (E) The related transcript levels of *WRKY41* in *wrky41* lines. Values are expressed as means ± SD (n=3). A significant difference analysis was Student's *t*-test (**, $p < 0.01$, ***, $p < 0.01$).

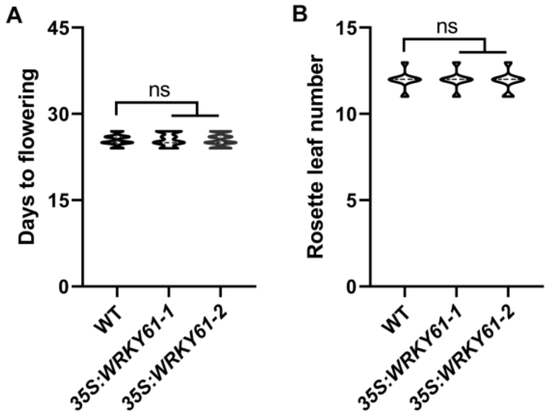


**Figure S5 The flowering phenotype of *35S:WRKY61*.** (A, B) The flowering phenotypes of *35S:WRKY61* were assessed by DTF (A) and RLN (B), respectively. Three biological replicates were counted with similar results, respectively. Values are expressed as means ± SD (n=15). A significant difference analysis was Student's *t*-test (ns, not significant).

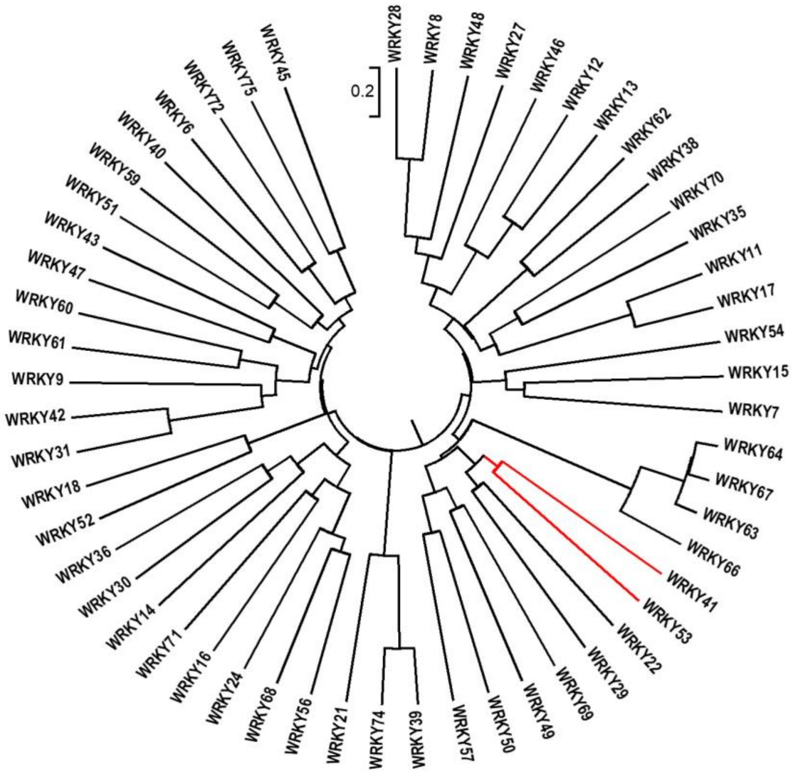


**Figure S6 Phylogenetic tree of *Arabidopsis* WRKY TFs**. The *Arabidopsis* WRKY genes were obtained in NCBI GenBank (https://www.ncbi.nlm.nih.gov/gene/). Then the phylogenetic tree was constructed using MEGA6 Software. Distance scale = 0.2. The red branch represents *WRKY41* and *WRKY53*.

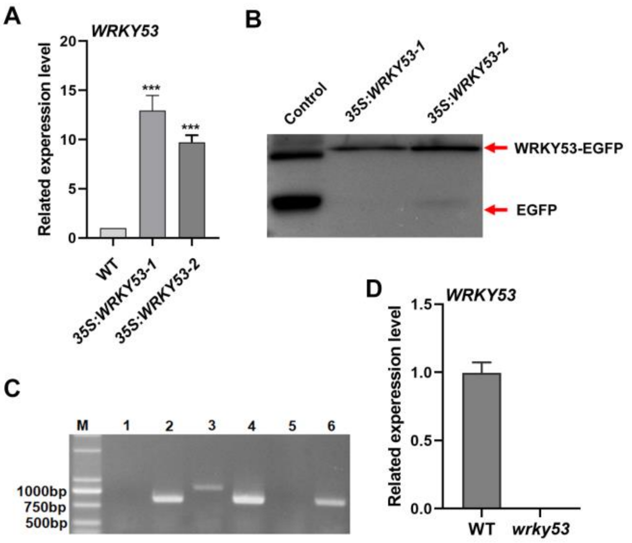


**Figure S7 Validation of *WRKY53* transgenic lines and T-DNA insertion mutants**. (A) The related transcript levels of *WRKY53* in two *35S:WRKY53* lines. Values are expressed as means ± SD (n=3). Similar results were obtained from three replicates. (B) Western blot analysis of two *35S:WRKY53*-GFP lines. Control, EGFP protein. (C) Validation of *WRKY53* T-DNA insertion mutants using the three primers. LP+BP included 1, 3, and 5 Lanes. BP+RP included 2, 4, and 6 Lanes. LP, RP: Left, Right genomic primer. BP: T-DNA border primer. M, DNA marker. 1-2, WT. 3-6, *wrky53* lines. Lanes 1-2, and 5-6 represent the homozygous lines; lanes 3-4 represent the heterozygous lines. (D) The related transcript levels of *WRKY53* in *wrky53* lines. A significant difference analysis was Student's *t*-test (***, $p < 0.01$).

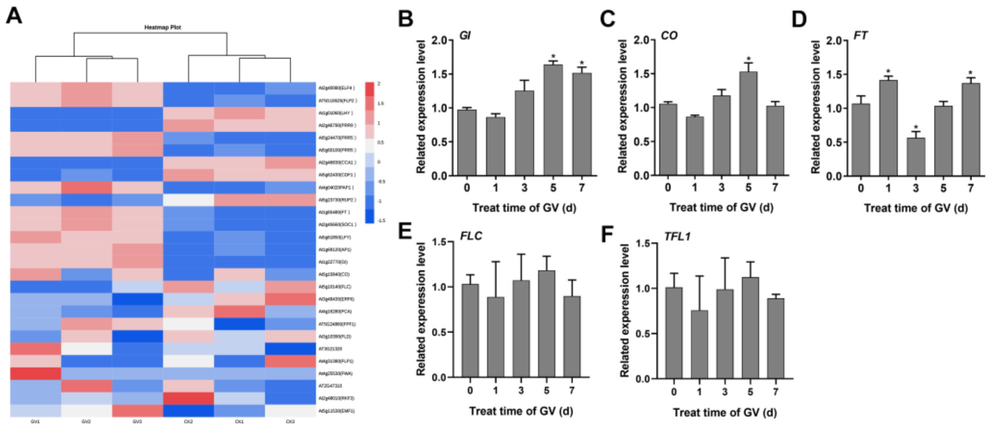


**Figure S8 Analysis and validation of flowering regulatory gene expression induced by GV**. (A) Clustering heat map of differentially expressed flowering-related genes induced by GV. (B-F) The relative expression level of the key flowering regulatory genes *GI* (B), *CO* (C), *FT* (D), *FLC* (E), and *TFL1* (F). Samples were collected at 0, 1, 3, 5, and 7 d after the treatment of 50 mg $l^{-1}$ GV and control (treated with 0 mg $L^{-1}$ GV). Each experiment was repeated three times with similar results. Values are expressed as means ± SD (n=3). A significant difference analysis was Student's *t*-test (*, $p < 0.05$).

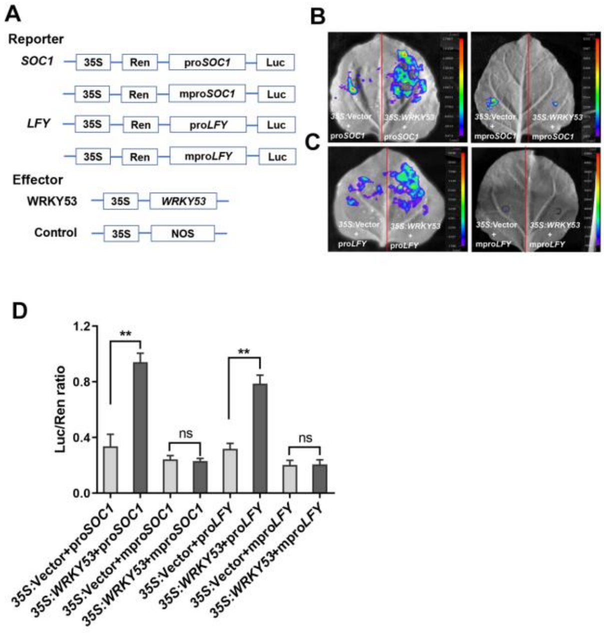


**Figure S9 WRKY53 directly activates the transcription of *SOC1* and *LFY*.** (A) The constructs were used for the transient transcriptional activity assay. The native and mutant promoters of *SOC1* and *LFY* were used as reporters, respectively. WRKY53 was used as an effector. (B) Transcription activation detection between WRKY53 and the pro*SOC1* (B left) and mpro*SOC1* (B right). (C) Transcription activation detection between WRKY53 and pro*LFY* (C left) and mpro*LFY* (C right). WRKY53 activated the expression of luciferase driven by the *SOC1* and *LFY* promoters. (D) Luc:Ren ratio after WRKY53 activated the transcription of *SOC1* and *LFY*. pro*SOC1*/pro*LFY*, the native promoter of *SOC1*/*LFY*. mpro*SOC1*/mpro*LFY*, the mutant promoter of *SOC1*/*LFY*. All experiments were repeated three times with similar results. Values are expressed as means ± SD (n=3). A significant difference analysis was Student's *t*-test (**, $p < 0.01$, ns, not significant).

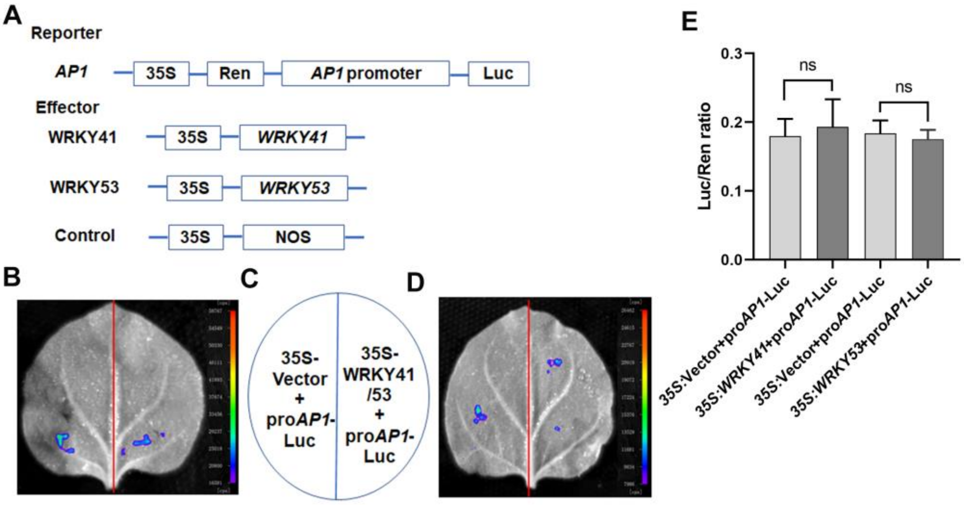


**Figure S10 WRKY41 and WRKY53 do not activate the transcription of *AP1*.** (A) The constructs were used for the transient transcriptional activity assay. The promoters of *AP1* were used as reporters. WRKY41 and WRKY53 were used as the effectors. (B, D) Transcription activation detection between WRKY41 (B) and WRKY53 (D) and *AP1* promoter by transient expression in *Nicotiana benthamiana*, respectively. (C) Distribution map of each treatment on *Nicotiana benthamiana* leaves. (E) Luc:Ren ratio detection after WRKY41 and WRKY53 acting on *AP1* promoter, respectively. All experiments were repeated three times with similar results. Values are expressed as means ± SD (n=3). A significant difference analysis was Student's *t*-test (ns, not significant).

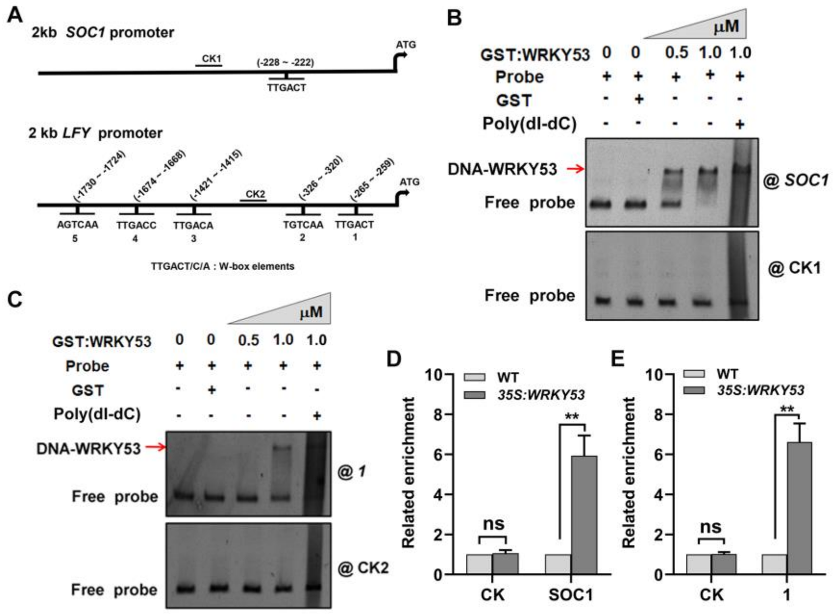


**Figure S11 WRKY53 directly binds to the *SOC1* and *LFY* promoters.** (A) The 2-kb promoters and *SOC1* and *LFY* fragments were used in EMSA. (-228~ -222), The position of the W-box in the *SOC1* promoter. 1, 2, 3, 4, and 5 represent the W-boxes located at (-265 ~ -259), (-326 ~ -320), (1421 ~ -1415), (-1674 ~ -1668), and (-1730 ~ -1724) bp in the *LFY* promoter. (B, C) GST-WRKY53 directly bound the W-box at (-228~ -222) bp in the *SOC1* promoter (B) and (-265 ~ -259) bp in the *LFY* promoter (C). 100-fold non-specific poly(dI-dC) was used to exclude non-specific binding between protein and probes. CK1 and CK2, negative control. (D, E) Enrichment of the W-box in the *SOC1* promoter (D) and 1 in the *LFY* promoter (E) as demonstrated by ChIP-qPCR. Samples were collected from three-week-old *35S:WRKY53* plants. The plus (+) and minus (-) symbols indicate the presence and absence of the indicated components. Arrows indicate band shifts. The triangle symbol indicates an increased concentration of GST-WRKY53. All experiments were repeated three times with similar results. Values are expressed as means ± SD (n=3). A significant difference analysis was Student's *t*-test (ns, not significant; **, $p < 0.01$).

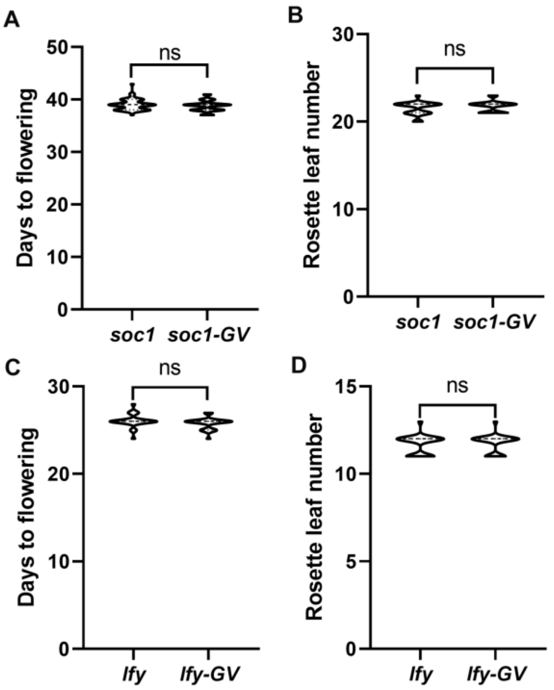


**Figure S12 The flowering phenotype of *soc1* and *lfy* mutants induced by GV**. (A, B) The flowering phenotypes of *soc1* treated without and with 50 mg $L^{-1}$ GV were assessed by DTF (A) and RLN (B), respectively. (C, D) The flowering phenotypes of *lfy* were assessed by DTF (C) and RLN (D) after 50 mg $l^{-1}$ GV-treated. 0 mg $L^{-1}$ GV treatment was used as a control. Three biological replicates were counted with similar results. Values are expressed as means ± SD (n=15). A significant difference analysis was Student’s t-test (ns, not significant).
