# Supplementary material for: The Transcription Factors WRKY41 and WRKY53 Mediate Early Flowering Induced by the Novel Plant Growth Regulator Guvermectin in Arabidopsis thaliana
Source: Int J Mol Sci. 2023 May 8;24(9):8424. doi: 10.3390/ijms24098424 (PMC10178944; doi:10.3390/ijms24098424)
Supplement: Supplementary file 1 [file ijms-24-08424-s001.zip › ijms-2317309-supplementary/ijms-2317309-Supplementary Tables.pdf]

**Supplementary Table 1**

Table S1 Flowering time of various plants in this study.

| Genotype | Days to flowering | Rosette leaf number |
|---|---|---|
| Long-day conditions | | |
| Col-0(WT) | 25.3±0.9 | 12.0±0.6 |
| *wrky41* | 25.2±0.8 | 12.1±0.5 |
| *35S:WRKY41-1* | 20.6±1.1 | 9.4±0.8 |
| *35S:WRKY41-2* | 20.4±1.1 | 9.4±0.7 |
| *wrky53* | 25.7±0.7 | 11.9±0.6 |
| *35S:WRKY53-1* | 21.1±0.9 | 9.3±0.7 |
| *35S:WRKY53-2* | 20.9±0.7 | 9.5±0.6 |
| *w41/w53-1* | 28.7±0.8 | 13.3±0.7 |
| *w41/w53-2* | 28.6±0.7 | 13.8±0.7 |
| *soc1* | 39.2±1.7 | 21.3±1.0 |
| *lfy* | 26.1±0.8 | 11.7±0.6 |
| *35S:WRKY41-lfy* | 26.0±0.7 | 11.9±0.5 |
| *35S:WRKY41-soc1* | 38.4±0.7 | 22.1±0.8 |
| *ahk2/3* | 26.0±0.5 | 12.1±0.7 |
| *ahk2/4* | 26.1±0.6 | 12.2±0.7 |
| *ahk3/4* | 26.1±0.8 | 12.4±0.6 |
| *35S:WRKY61-1* | 25.5±1.1 | 12.0±0.5 |
| *35S:WRKY61-2* | 25.1±1.1 | 11.9±0.6 |
| GV treatment | | |
| Col-0 (WT) | 21.4±1.3 | 9.9±0.7 |
| *wrky41* | 23.3±0.9 | 11.8±0.6 |
| *wrky53* | 22.9±1.0 | 12.0±0.5 |
| *w41/w53-1* | 28.2±0.7 | 13.7±0.7 |
| *w41/w53-2* | 28.4±0.7 | 13.9±0.9 |
| *soc1* | 39.1±1.8 | 21.6±0.9 |
| *lfy* | 25.8±0.7 | 11.8±0.5 |
| *ahk2/3* | 22.2±0.7 | 9.4±0.7 |
| *ahk2/4* | 21.9±0.8 | 9.4±0.8 |
| *ahk3/4* | 22.1±0.8 | 9.3±0.8 |
| Short-day conditions | | |
| Col-0 (WT) | 66.4±0.9 | 46.0±0.9 |
| GV treatment | | |
| Col-0 (WT) | 58.1±1.0 | 39.8±1.1 |

Flowering time is presented as the days to flowering and the rosette leaf number. Values are mean ± SD. from at least 45 plants for each genotype.

**Supplementary Table 2**

Table S2 The transcription factors induced by GV.( log2FoldChange>1.5)

| Gene id | Locus Names | Gene_name | $log_2$FoldChange |
|---|---|---|---|
| 838467 | At1g18860 | WRKY61 | 4.154944 |
| 826708 | At4g11070 | WRKY41 | 3.284362 |
| 816726 | At2g21900 | WRKY59 | 3.129090 |
| 831147 | At5g13080 | WRKY75 | 2.731508 |
| 819248 | At2g46400 | WRKY46 | 2.033432 |
| 836602 | At5g64810 | WRKY51 | 1.943350 |
| 844423 | At1g80840 | WRKY40 | 1.834195 |
| 825775 | At4g04450 | WRKY42 | 1.790545 |
| 831826 | At5g10900 | WRKY62 | 1.741529 |
| 844398 | At1g80590 | WRKY66 | 1.729935 |
| 818669 | At2g40740 | WRKY55 | 1.645343 |
| 828481 | At4g23810 | WRKY53 | 1.632624 |
| 825435 | At3g62610 | MYB11 | 5.033301 |
| 842957 | At1g66390 | MYB90 | 3.881552 |
| 842955 | At1g66370 | MYB113 | 3.755247 |
| 819332 | At2g47190 | MYB2 | 3.125961 |
| 839219 | At1g26780 | MYB117 | 2.755653 |
| 821904 | At3g23250 | MYB15 | 2.194089 |
| 841218 | At1g48000 | MYB112 | 1.773851 |
| 843804 | At1g69020 | MYB31 | 1.645950 |
| 818902 | At2g43000 | NAC042 | 3.159705 |
| 832299 | At5g22380 | NAC090 | 2.588656 |
| 841722 | At1g52890 | NAC019 | 2.131126 |
| 819562 | At3g04070 | NAC047 | 1.908843 |
| 840317 | At1g34180 | NAC016 | 1.699634 |
| 816209 | At2g17040 | NAC036 | 1.607708 |

| | | | |
|---|---|---|---|
| 820512 | At3g13220 | ABCG26 | 4.988130 |
| 825269 | At3g60970 | ABCC15 | 3.993031 |
| 821902 | At3g23240 | ERF1 | 3.880447 |
| 835447 | At5g53660 | GRF7 | 1.624781 |

**Supplementary Table 3**

Table S3 List of primers used in the experiments.

| Primer | 5’--------3’ |
|---|---|
| qPCR primer | |
| UBQ10-F | CACACTCCACTTGGTCTTGCGT |
| UBQ10-R | TGGTCTTTCCGGTGAGAGTCTTCA |
| GI-F | AGTCGTTGCTTATGTTGA |
| GI-R | GCTTCTCAGTTGATGGAT |
| FT-F | CTACAACTGGAACAACCTT |
| FT-R | AACACGACACGATGAATT |
| CO-F | ATAGAGTTGTTCCGCTTA |
| CO-R | ATTGTCGTTGTAGTGAGT |
| TSF-F | ATTCATCGTATTGTGTTG |
| TSF-R | AAGACCAAGATTGTAGAT |
| SOC1-F | ATGAAGAGAATAGAGAATG |
| SOC1-R | TTAGTATGCCTCAGATAA |
| LFY-F | AGATAGCGGAGTTAGGTT |
| LFY-R | GACTATTCATCATCTCTTCAAG |
| AP1-F | AAGAGGATAGAGAACAAG |
| AP1-R | AAGAATCAGTGGAGTATT |
| WRKY41-F | CAGCTTCAGGGTTCGTCTTC |
| WRKY41-R | ATCAGGCGCAAGCTCTATCG |

| | |
|---|---|
| WRKY53-F | GACGGGGATGCTACGGTTT |
| WRKY53-R | TTTTGGGTAATGGCTGGTTTG |
| WRKY61-F | GTGCAGCTTACGGCAACATT |
| WRKY61-R | GGATCGAAAGATGACCCGGC |
| WRKY59-F | TCACAAGTGTTCGAGCCCAG |
| WRKY59-R | CAGAAGGGCTTGGGTGGTTA |
| WRKY46-F | CATCACATCCCCGAAGACGA |
| WRKY46-R | TTCGGACTTGGTCGGTTTCA |
| WRKY75-F | ATATGGCCAAAAGGCCGTCA |
| WRKY75-R | TGCTCGAAGTTTTCGGTGGA |
| WRKY55-F | GTCGTCTCAAGGAACGGAGG |
| WRKY55-R | AGCGCCGGATCTTTCTACTG |
| WRKY66-F | AGACCATCAAAACCTCTCCGC |
| WRKY66-R | TCGCAGTTTTGGTCTTTCGC |
| WRKY62-F | CAACCCCGATCTACCACGAC |
| WRKY62-R | CTGGTCCTTGGTGTAGGCAC |
| WRKY42-F | GTCCTGTTCGCAAGCAAGTG |
| WRKY42-R | ATGTTCATAGCCGCAGGAGG |
| WRKY40-F | AACCGCCACATCTCTCATGG |
| WRKY40-R | TGGGCTCGTCACTTTCTTCG |
| WRKY51-F | ACGGGTCATCGAGTTGCATT |
| WRKY51-R | TCACCGAGCAACCTTCACTT |
| FLC-F | GGCTAGCCAGATGGAGAATAATCA |
| FLC-R | CACCGGAAGATTGTCGGAGA |
| TFL1-F | CCAAGGCCAAGCATAGGGAT |
| TFL1-R | CCAAGATCATACTCGACCGCA |
| BCS1-F | GAGATCAAAGAGCACCGCCT |
| BCS1-R | AGGCACACATGGAAGACGTT |
| CYP703A2-F | AGGCACACATGGAAGACGTT |

| | |
|---|---|
| CYP703A2-R | CGGTTGCTTGATCGCTTCAG |
| ERF1-F | CCAGGACACGGGGAAGTATG |
| ERF1-R | TCACAGTTTCACCAGTCGCA |
| JRG21-F | TGACCATTCTCTTGCCGGAC |
| JRG21-R | ATGGAAAATAAAACAGTCACCTGAA |
| LBD1-F | ACTATTGCACACCGCGTCTT |
| LBD1-R | GGCACCTGCACATCCATACA |
| PR1-F | ACGCAGAACAACTAAGAGGCA |
| PR1-R | ACCCCAGGCTAAGTTTTCCC |
| Mutant verification primer | |
| LBa1.3 | ATTTTGCCGATTTCGGAAC |
| *wrky41*- LP | GAAAGGTTCCAGGATCTCCAG |
| *wrky41*-RP | GGGGAAGCCTGTGTTAATCTC |
| *wrky53*-LP | TCAGGCACGACTTAGAGAAGC |
| *wrky53*-RP | GGGAAAGTTGTGTCAATCTCG |
| CRISPR-Cas9 guide oligo | |
| LFY-oligo 1 | GATTGAGACGATTGCAAGAAGAGG |
| LFY-oligo 2 | AAACCCTCTTCTTGCAATCGTCTC |
| WRKY41-oligo 1 | GATTGTCTCAACAAATACTTCCAC |
| WRKY41-oligo 2 | AAACGTGGAAGTATTTGTTGAGAC |
| WRKY53-oligo 1 | GATTGGCCATTACCCAAAAGCCAA |
| WRKY53-oligo 2 | AAACTTGGCTTTTGGGTAATGGCC |
| SOC1-oligo 1 | GATTGAGTGACTTTCTCCAAAAGA |
| SOC1-oligo 2 | AAACTCTTTTGGAGAAAGTCACTC |
| CRISPR-Cas9 sequence primer | |
| WRKY41-F | GATCCCACTTCCAGAGAAACGAGAAAC |
| WRKY41-R | TTAATCTCAAGAATATTCAATACTGTACTAC |
| LFY-F | GACTTGGTGGTTTAGAGGGACTATT |
| LFY-R | GAAAGTCGGG CATAGAAATGTTGAGAA |

| | |
|---|---|
| WRKY53-F | CAGATGCACACATCGTAGCACACAAA |
| WRKY53-R | CTCTCGCTGATCATTACTTACTAGTTACT |
| SOC1-F1 | ACCTACTCTCTTTGATTCATATATGAAATG |
| SOC1-R1 | ATAAGAGGGTATATGTACTTGATACTAC |
| GST tag protein primer | |
| WRKY53-F | CGGAATTC ATGTTACCAAAGTGGTCAGA |
| WRKY53-R | CCGCTCGAGTTATGGCTTT TGGGTAATGG |
| WRKY41-F | ACGCGTCGACCGATGTTGCCAAAGTGGACAGAG |
| WRKY41-R | CCGCTCGAGTTAGGCCCTGAGATTCTGGT |
| EMSA primer | |
| EMSA-LFY-1-F | GAAACTGAGGGAGTGAGACAGTCAAAATAAAACAGACTGAGAGCA |
| EMSA-LFY-1-R | TGCTCTCAGTCTGTTTTATTTTGACTGTCTCACTCCCTCAGTTTC |
| EMSA-LFY-2-F | TCTTTCTGTTATTTAATAAATTTGACATTACCAAAAAAAAATTT |
| EMSA-LFY-2-R | AAATTTTTTTTTGGTAATGTCAAATTTATTAAATAACAGAAAGA |
| EMSA-LFY-3-F | TTTATATTCCTACGTGTCAAATTATGAATGGTCATAATCAGAGTAGAGAA |
| EMSA-LFY-3-R | TTCTCTACTCTGATTATGACCATTCATAATTTGACACGTAGGAATATAAA |
| EMSA-LFY-4-F | TGTCGATGAAAGAAAAAGTTTGACTTGTTAAGTCCCAACTGTC |
| EMSA-LFY-4-R | GACAGTTGGGACTTAACAAGTCAAACTTTTTCTTTCATCGACA |
| EMSA-LFY-5-F | ACTTGTTAAGTCCCAACTGTCAATTTCCCAGCAAGACACAT |

| | |
|---|---|
| EMSA-LFY-5-R | ATGTGTCTTGCTGGGAAATTGACAGTTGGG ACTTAACAAGT |
| EMSA-SOC1- F | TGAAAGGAGGTTGCATCCTTCA |
| EMSA-SOC1- R | GGTAGATCAATGGTGCAACCA |
| EMSA-SOC1-CK-F | ACACACAAATAGATGAAACGAGG |
| EMSA-SOC1-CK-R | CGATTGGCTAAAAGGGTTTGATA |
| Luc primer | |
| SOC1-Luc-F | AACTGCAGCCTGAAGCATAGCCTGGTTCTA |
| SOC1-Luc-R | CGGGATCCTCAATGGTGCAACCACCTCA |
| LFY-Luc-F | AACTGCAGTGTGAAAATGATACTGAGAAAA TAGG |
| LFY-Luc-R | CGGGATCCTGTAAAAATGACCACTCGAGCA AC |
| W41-SK-F | GCTCTAGAATGTTGCCAAAGTGGACAGAG |
| W41-SK-R | CGGAATTCTTAGGCCCTGAGATTCTGGT |
| W53-SK-F | GCTCTAGAATGTTACCAAAGTGGTCAGA |
| W53-SK-R | CGGAATTCTTATGGCTTTTGGGTAATGG |
